# A Zwitterionic Copolymer at High Temperature and High Salinity for Oilfield Fracturing Fluids

**DOI:** 10.3390/polym17202733

**Published:** 2025-10-12

**Authors:** Bo Jing, Yuejun Zhu, Wensen Zhao, Weidong Jiang, Shilun Zhang, Bo Huang, Guangyan Du

**Affiliations:** 1 State Key Lab of Offshore Oil & Gas Exploitation, Beijing 100028, China; jingbo@cnooc.com.cn (B.J.); zhuyj3@cnooc.com.cn (Y.Z.); zhaows@cnooc.com.cn (W.Z.); zhangshl53@cnooc.com.cn (S.Z.); huangbo@cnooc.com.cn (B.H.); 2 CNOOC Research Institute Ltd., Beijing 100028, China; 3 China National Offshore Oil Corporation, Beijing 100010, China; jiangwd@cnooc.com.cn; 4 State Key Laboratory of Advanced Separation Membrane Materials, Zhejiang Key Laboratory of Advanced Polymer Materials Modification and Application Technology, College of Materials Science and Engineering, Zhejiang University of Technology, Hangzhou 310014, China

**Keywords:** zwitterionic copolymer, fracturing fluids, high temperature and high salinity, sand-carrying

## Abstract

With the increasing exploration and development of deep shale gas resources, water-based fracturing fluids face multiple challenges, including high-temperature resistance, salt tolerance, and efficient proppant transport. In this study, a zwitterionic polymer (polyAMASV) is synthesized via aqueous two-phase dispersion polymerization, using acrylamide (AM), 2-acrylamido-2-methylpropanesulfonic acid (AMPS), acrylic acid (AA), stearyl methacrylate (SMA), and 4-vinylpyridine propylsulfobetaine (4-VPPS) as monomers. The introduction of hydrophobic alkyl chains effectively adjusts the viscoelasticity of the emulsion, while the incorporation of zwitterionic units provides salt tolerance through their intrinsic anti-polyelectrolyte effect. As a result, the solutions of such copolymers exhibit stable apparent viscosity in both NaCl and CaCl_2_ solutions and under high temperatures. Meanwhile, polyAMASV outperforms conventional samples across various saline environments, reducing proppant settling rates by approximately 20%. Moreover, the solutions exhibit rapid gel-breaking and low residue characteristics, ensuring effective reservoir protection. These results highlight the promising potential of polyAMASV for deep shale gas fracturing applications.

## 1. Introduction

Fracturing fluid is a critical component in the process of shale gas development [1], enabling pressure transmission, formation fracturing, cooling, and lubrication [2,3]. Among the various types of fracturing fluids, water-based systems that contain water [4], drag reducers, and other chemical additives such as fluid loss control agents, blocking agents, lubricants, and inhibitors are widely used in large-scale hydraulic fracturing operations due to their environmental compatibility and cost-effectiveness [5]. Drag reducers play a critical role in enhancing flow efficiency by reducing turbulence, altering boundary layer behavior [6], and promoting the formation of associated network structures through extended polymer chains [7,8]. Simultaneously, the oil–water two-phase flow behavior at the pore scale also directly influences efficiency. Therefore, the molecular structure of the drag reducers and the viscosity of their solutions, as well as the suspension stability of proppants, are crucial for the performance of fracturing fluids [9]. Therefore, the design and synthesis of drag reducers with high performance have attracted widespread attention from researchers in both academia and the petroleum engineering industry.

Most drag reducers are typically polymer-based materials [10], including natural polymers (e.g., xanthan gum, guar gum, and starch), artificially modified natural polymers, and synthetic polymers [11,12,13]. These materials have been widely used in unconventional reservoir fracturing operations, such as shale gas, due to their advantages of abundant availability, low cost, and environmental friendliness [14]. However, as the global oil and gas industry advances toward deep and ultra-deep shale gas exploration and development, extreme conditions such as high temperature and high salinity impose more stringent performance requirements on drag reducers [15,16]. Taking the natural polymer, guar gum, as an example [17], the glycosidic bonds in its molecular chains are prone to hydrolytic cleavage under high temperatures, while the chains tend to coil and deactivate in high-salinity environments, leading to a significant decline in drag-reducing performance. Consequently, such materials struggle to meet the engineering demands of deep shale gas development [18].

Synthetic drag reducers, particularly acrylamide-based polymers, have attracted significant attention due to their designable molecular structures and controllable molecular weight/chain architecture [19,20,21]. Through strategies such as introducing sulfonic groups, ring structures, or copolymerization modification, these materials have demonstrated remarkable improvements in high-temperature resistance, showing distinct advantages for deep shale gas extraction [22]. For instance, Zhang et al. developed an acrylamide/acrylic acid/cetyl methyldiallyl ammonium chloride terpolymer with a temperature tolerance of 120 °C [23], while Chen et al. synthesized an acrylamide/acrylic acid/sodium p-styrenesulfonate/cetyl dimethyldiallyl ammonium chloride quaternary copolymer capable of withstanding temperatures up to 130 °C [24]. However, in high-salinity environments, the inorganic salt ions shield the charge repulsion, causing them to coil, particularly in the presence of divalent cations; this can lead to precipitation and flocculation of high-charge-density polymers [25]. This prevents the chains from extending into the turbulent boundary layer to suppress vortex development, thereby impairing drag-reducing performance and reducing operational efficiency. Moreover, chain contraction collapses the solution’s three-dimensional network structure, leading to proppant settling under gravity, which may cause sand blockage and diminished fracture conductivity [26]; its flow behavior within fractures is inherently a complex three-dimensional multiphase flow process. The efficiency not only depends on the molecular properties of the drag-reducing agent, but is also influenced by secondary flows and mixing effects induced by the geometry of the flow channels (such as bends and branch structures) [27]. Consequently, enhancing the salt tolerance of fracturing fluids remains a critical challenge for deep shale gas development. Notably, zwitterionic polymers that feature both cationic and anionic groups on their molecular chains exhibit a unique anti-polyelectrolyte effect, where increasing salt concentration promotes chain extension [28]. This property allows them to maintain sufficient chain extension and solution viscosity, even in high-salinity environments, offering a novel solution to the failure of conventional synthetic polymers under high-temperature/high-salinity conditions [29].

In this study, a zwitterionic copolymer (polyAMASV) containing hydrophobic long chains is synthesized via aqueous two-phase dispersion polymerization using 4-vinylpyridine propyl sulfobetaine as the zwitterionic monomer. The polymerization conditions and the properties of the solution properties of the resultant copolymer are systematically investigated. A series of rheological tests demonstrate that the presence of zwitterionic components endows the copolymer solution with significantly improved temperature resistance, salt tolerance, and shear recovery properties. Moreover, owing to its clean synthesis approach, the polymer combines rapid degradability with low residue characteristics, showing dual potential for both reservoir protection and shale gas fracturing applications.

## 2. Results and Discussion

**Figure 1:**
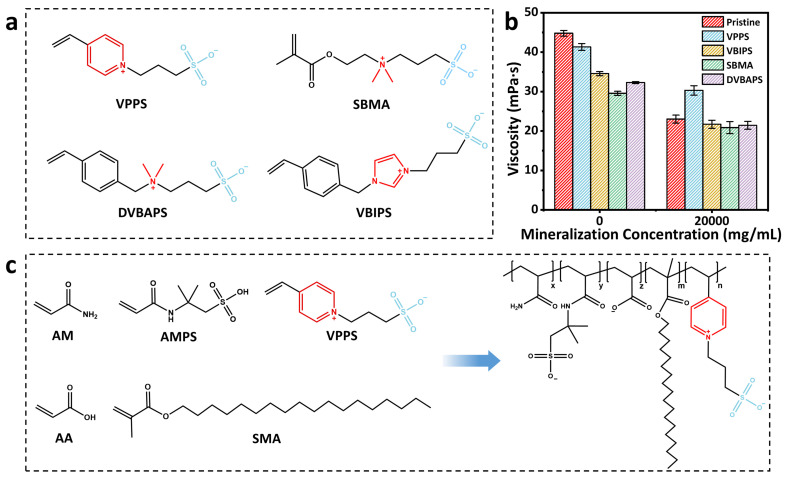
(**a**) Molecular formulas of various zwitterionic monomers. (**b**) The viscosity changes in different zwitterionic polymers in water and saline solutions. (**c**) Description of synthesis process of polyAMASV.

To develop temperature- and salt-resistant polyacrylamide copolymers, this study systematically screens various zwitterionic monomers with molecular structures, as shown in Figure 1a. All the zwitterionic monomers with same molar ratio were introduced into the copolymer, and then the solutions with the same concentration of this copolymer were prepared. The copolymer without zwitterionic monomers was also synthesized for comparison. 

The results of viscosity measurement on these solutions are shown in Figure 1b. It is shown that the solution of the copolymer without zwitterionic monomers exhibits the highest viscosity in aqueous solution, while the solutions of copolymers containing zwitterionic monomers show relatively low viscosity. This phenomenon is likely due to the fact that the introduction of zwitterionic monomers weakens the hydrophobic effect and then reduces the viscosity. However, under high-salinity conditions (20 g/L), the solutions of copolymers with zwitterionic monomer show comparable or even higher viscosity compared to that of the copolymer without zwitterionic monomers [30]. When comparing different zwitterionic monomers, we can find that 4-VPPS modified system demonstrates optimal overall performance by balancing the synergistic effects between hydrophobic associations and ionic stability (Figure 1c).

**Figure 2:**
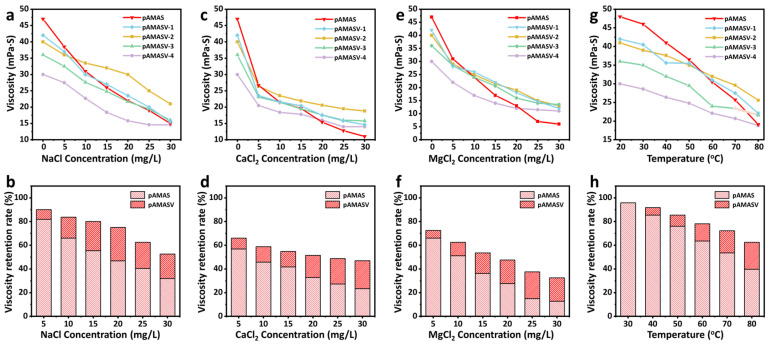
Viscosity changes and viscosity retention rate of polymer at (**a**,**b**) NaCl solution, (**c**,**d**) CaCl_2_ solution, (**e**,**f**), MgCl_2_ solution, and (**g**,**h**) different temperatures.

The zwitterionic content significantly influences performance. Appropriate incorporation enhances chain rigidity and promotes microphase separation, while excess amounts cause structural damage. A series of polymer emulsions (polyAMASV1–4) were prepared by progressively increasing VPPS content, among which polyAMASV-2 exhibited the most superior overall performance. In NaCl solutions, the viscosity loss rate remained consistently lower than the unmodified polyAMAS, with the viscosity exceeding that of polyAMAS when salt concentration surpassed 10 g/L. PolyAMASV-2 maintained higher viscosity retention, even under the strong charge screening effect of Ca^2+^/Mg^2+^ (Figure 2a–f). Additionally, the temperature-dependent viscosity behavior is demonstrated in Figure 2g–h, confirming that incorporation of VPPS enhances thermal stability in the systems. These results indicate that VPPS can simultaneously improve both salt resistance and temperature tolerance. However, excessive addition of VPPS will reduce the intrinsic viscosity of the system, while the VPPS content of polyAMASV-2 reaches the optimal balance. Therefore, the subsequent research subjects that have been emphasized are all polyAMASV-2 systems.

**Figure 3:**
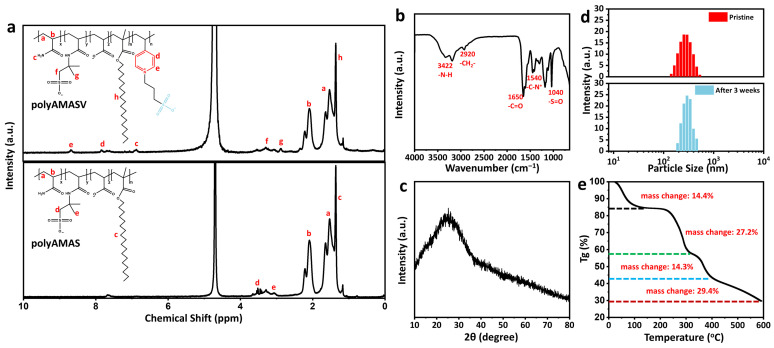
(**a**) ^1^H NMR spectra of polyAMASV and polyAMAS. (**b**) FTIR and (**c**) XRD spectra of polyAMASV. (**d**) The size distribution of polyAMASV. (**e**) TGA of polyAMASV.

The synthesis of the copolymer is confirmed by the ^1^H NMR and FT-IR spectra. As shown in Figure 3a, the absence of peaks in the 5–6 ppm region and the appearance of peaks at a, b indicate the complete polymerization of the C=C bonds [28]. The signal at c confirms the presence of the -NH_2_ group, demonstrating the successful polymerization of acrylamide [31]. Peaks at d and e signify the appearance of pyridyl signals, confirming the successful incorporation of VPPS. The signals at f–g demonstrate the introduction of AMPS into the system. Finally, a characteristic peak of long alkyl chains appears at h, providing evidence for the incorporation of hydrophobic monomers. A comparison of the NMR spectra of the polyAMASV and polyAMAS reveals that the spectrum of polyAMAS, which lacks the incorporated VPPS component, does not exhibit the characteristic pyridine peaks in the 7.8–8.5 ppm region [32]. Additionally, FTIR spectroscopy demonstrated the disappearance of the C=C stretching vibration, suggesting the successful copolymerization of the monomers (Figure 3b) [33]. XRD analysis (Figure 3c) reveals a broad diffraction peak at 2θ = 20–30°, indicating the amorphous structure of polyAMASV, which contributes to its excellent thickening capability and water solubility. Particle size analysis (Figure 3d) showed a narrow distribution with an average diameter of 255 nm and 70% of particles ranging 250–350 nm, ensuring good storage stability and rheological performance. The particle size of the samples that had been left stationary for three weeks was further tested and found to be relatively stable. The TGA results (Figure 3e) demonstrate that the copolymer exhibits excellent thermal stability, with a weight loss of 14.4% before 220 °C. Free water and bound water in the polymer evaporate as temperature increases. Between 220 °C and 345 °C, the weight loss reaches 27.2%, as the amide groups and hydrophobic side chains in the polymer decompose at high temperatures. Between 345 °C and 420 °C, the decomposition of the carbon skeleton leads to further mass loss, with a mass loss of 14.34%. Finally, the residual components and impurities of the polymer begin to decompose (420–600 °C), resulting in a mass loss of 14.34%. This thermal stability shows the requirements of high-temperature conditions in deep shale gas applications.

**Figure 4:**
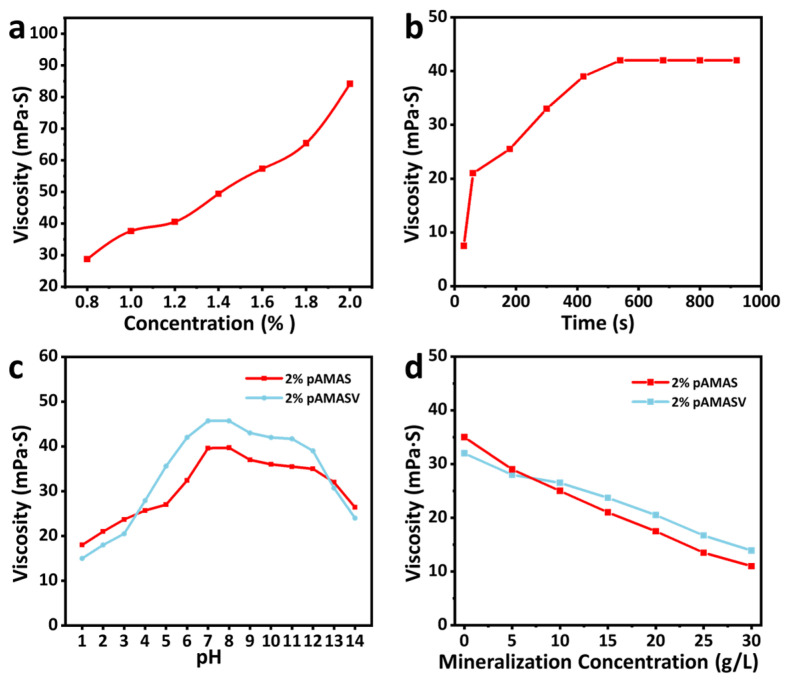
(**a**) Different concentrations, (**b**) solution time, (**c**) salt resistance, and (**d**) pH resistance of the polyAMASV solution viscosity.

The apparent viscosity and dissolution time are fundamental parameters of fracturing fluids, determining operational efficiency and safety. As shown in Figure 4a, the solution viscosity increases with polymer concentration, demonstrating typical polymer solution behavior. This occurs because higher polymer concentrations enhance intermolecular interactions, increasing flow resistance and, consequently, viscosity [34]. Preparing a 1.2 wt% polyAMASV polymer solution, the system achieves viscosity equilibrium within 450 s (Figure 4b), indicating the complete dissolution and formation of a stable solution. Comprehensive salt tolerance and acid–alkali resistance tests were conducted on the polyAMASV emulsion. Figure 4c shows that the apparent viscosity of polyAMASV solution exhibits a three-stage variation with increasing salinity: (1) rapid decrease at 0–5 g/L, due to Na^+^ shielding of -SO_3_^−^ groups; (2) slower decline at 5–15 g/L, attributed to enhanced hydrophobic associations; and (3) only 37.5% viscosity reduction at 20 g/L, significantly better than the polyAMAS system (54%), benefiting from the anti-polyelectrolyte effect of zwitterionic groups. Figure 4d demonstrates stable viscosity maintenance in the pH 6–13 range. However, under strong acidic (pH < 6) or alkaline (pH > 13) conditions, viscosity decreases by 35% and 30%, respectively, due to amide group hydrolysis/charge neutralization and ester hydrolysis, yet still outperforms the polyAMAS system. These results indicate that polyAMASV possesses excellent salt tolerance and moderate acid/alkali stability, making it particularly suitable for weakly acidic/alkaline environments.

**Figure 5:**
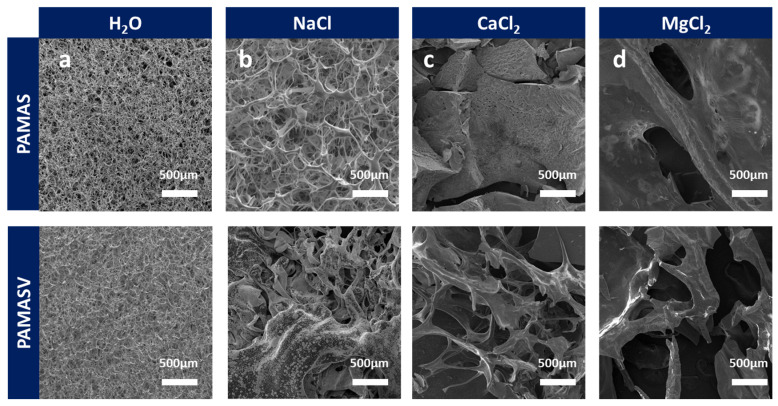
Microstructure changes in polyAMASV in (**a**) H_2_O (**b**) NaCl (**c**) CaCl_2_, and (**d**) MgCl_2_.

To address the regional heterogeneity of ionic composition in shale gas reservoir brines, SEM is systematically employed to analyze the effects of different salt solutions on polymer network structures. Figure 5a reveals that both emulsions formed cross-linked three-dimensional network structures in the aqueous solution. In the NaCl solution, polyAMASV’s zwitterionic groups partially counteracted the charge shielding effect, resulting in relatively minor structural damage (Figure 5b). Under CaCl_2_ conditions, polyAMAS completely disintegrated due to Ca^2+^-COO^−^ chelation, whereas polyAMASV maintained most network integrity through its rigid structure that inhibited excessive polymer chain folding (Figure 5c). In the MgCl_2_ environment, both polymer networks suffered irreversible damage due to Mg^2+^ high charge density (Figure 5d). These results demonstrate polyAMASV’s structural stability advantages in Na^+^/Ca^2+^-dominated formation waters.

**Figure 6:**
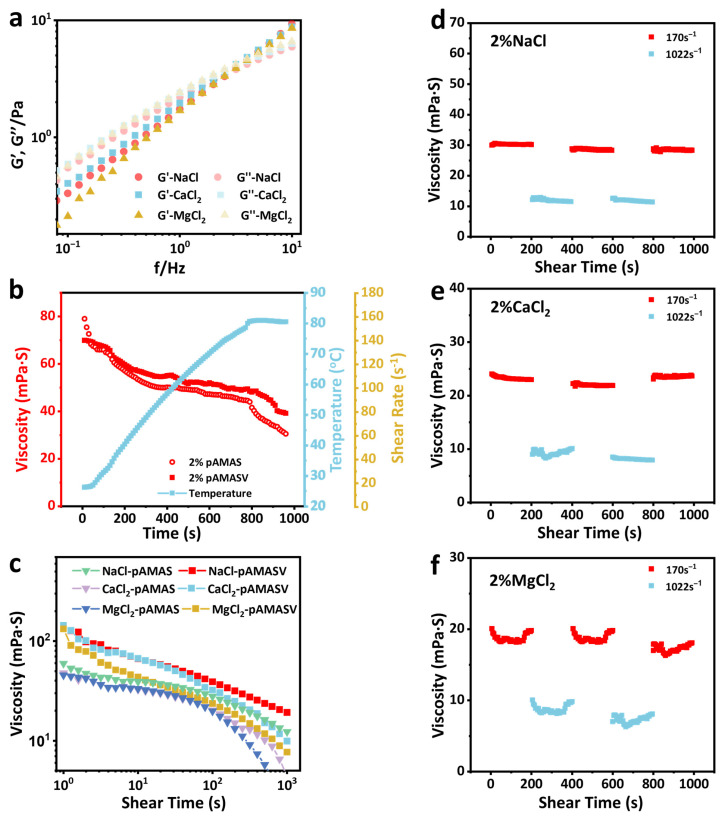
(**a**) Frequency scan test, (**b**) temperature resistance test, (**c**) shear resistance test in salt solution, (**d**) shear stability test in NaCl solution, (**e**) shear stability test in CaCl_2_ solution, and (**f**) shear stability test in MgCl_2_ solution.

The dynamic mechanical behavior of polyAMASV solutions is investigated through rheological testing. Frequency sweep analysis (Figure 6a) demonstrated that in all saline systems, both the storage modulus (G′) and loss modulus (G″) increased in frequency, ultimately exhibiting G′ exceeding G″’s characteristic behavior of cross-linked networks. Notably, the modulus variations in Na^+^ and Ca^2+^ systems showed similar trends, while the Mg^2+^ system displayed reduced moduli due to its higher charge density. Temperature sweep results (Figure 6b) demonstrated that polyAMASV exhibited a 30% smaller viscosity reduction at 80 °C compared to polyAMAS [33]. After the introduction of VPPS monomers, zwitterionic ions can form a more stable network structure through intramolecular and intermolecular electrostatic attraction, demonstrating a certain degree of temperature resistance. Shear rate tests (Figure 6c) indicated shear-thinning behavior for polyAMASV across different saline solutions, though with consistently smaller viscosity reductions than polyAMAS. Shear recovery experiments (Figure 6d–f) showed that in the NaCl solution, the polymer maintained stable viscosity under both high and low shear rates. In the CaCl_2_ solution, Ca^2+^ influenced molecular chain dynamics, causing fluctuations during entanglement network reorganization. Mg^2+^ induced significant chain rearrangement, leading to dramatic viscosity variations and reduced stability. These results collectively confirm the exceptional resistance of polyAMASV to Na^+^ and Ca^2+^ environments.

**Figure 7:**
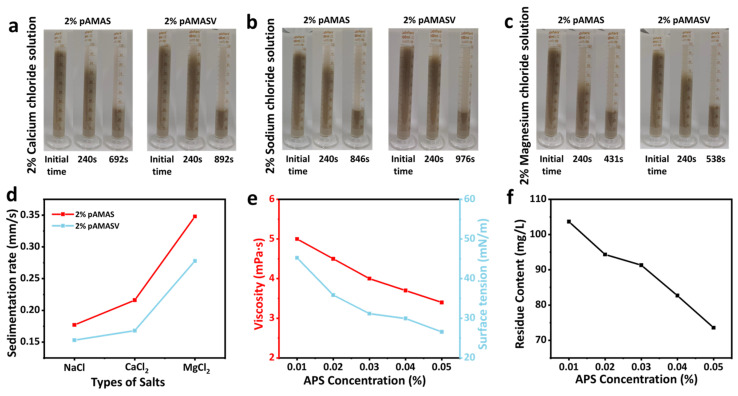
Static suspended sand test of polyAMAS and polyAMASV in (**a**) NaCl (**b**) CaCl_2_ (**c**) MgCl_2_ solution. (**d**) Sedimentation rate. (**e**) Viscosity and the surface tension of gel breaking fluids. (**f**) Residue content of gel breaking fluids.

Building upon polyAMASV’s exceptional viscoelastic performance in saline solutions, we systematically investigated its sand-carrying characteristics. At a 2 wt% concentration, polyAMASV demonstrated 30%, 28%, and 15% reductions in proppant settling rates compared to polyAMAS in the NaCl, CaCl_2_, and MgCl_2_ solutions, respectively. This improvement is attributed to its zwitterionic structure maintaining extended molecular chain conformations through charge balance (Figure 7a–c). The highest settling rate in the MgCl_2_ solution corresponded with the observed rheological performance decline (Figure 7d).

During the flowback phase of shale gas fracturing operations, fracturing fluid breaking is essential, with broken fluid properties significantly impacting reservoir protection and production efficiency. Breaking tests (Figure 7e) revealed that with 0.05 wt% ammonium persulfate addition, the solution’s apparent viscosity decreased to 3 mPa·s while maintaining stable surface tension at 34.286 mN/m, with residual content as low as 74 mg/L, confirming complete breaking capability (Figure 7f). This system simultaneously meets both proppant-carrying performance and breaking requirement specifications.

## 3. Conclusions

In conclusion, we successfully prepared polyacrylamide emulsion polyAMASV via aqueous two-phase polymerization using acrylamide (AM), 2-acrylamido-2-methylpropanesulfonic acid (AMPS), acrylic acid (AA), stearyl methacrylate (SMA), and 4-vinylpyridine propyl sulfobetaine (4-VPPS) as monomers. Based on a zwitterionic protection–hydrophobic association enhancement synergistic mechanism, the material achieved combined salt/temperature resistance and viscosity-enhancing properties. The TGA analysis confirmed its exceptional thermal stability, meeting the requirements for high-temperature reservoir applications. Rheological tests demonstrated storage moduli of 9.29 Pa (NaCl), 8.76 Pa (CaCl_2_), and 6.29 Pa (MgCl_2_) in three saline solutions—all exceeding loss moduli and exhibiting elastic-dominated rheological behavior—with corresponding proppant settling rate reductions of 18%, 28%, and 15%. This work not only provides a novel strategy for developing high-performance zwitterionic copolymer drag reducers, but also opens new avenues for designing fracturing fluids for deep shale gas reservoirs.

## 4. Materials and Methods

### 4.1. Materials

Acrylamide (AM 99.9%), 2-acrylamide-2-methylpropanesulfonic acid (AMPS 98.0%), acrylic acid (AA 99.0%), stearyl methacrylate (SMA 96.0%) and 2,2′-azobis [2-methylpropionamidine] dihydrochloride (V-50 98.0%) were purchased from Aladdin Industrial Corporation (Shanghai, China). Benzene (99.5%), 4-vinylpyridine (4-VP, 99.9%), 1,3-propanesultone (1,3-PS, 99%), and acetone (99%) were purchased from Shanghai Macklin Biochemical Co., Ltd. (Shanghai, China). Dodecyl mercaptan (98.0%), octylphenol polyethylene glycol ether (OP-10 99.0%) and sodium hydroxide (96.0%) were purchased from Shanghai Titan Technology Co., Ltd. (Shanghai, China). Ammonium sulfate (AS 99.9%), sodium chloride (NaCl), calcium chloride (CaCl_2_) and magnesium chloride (MgCl_2_) were all sourced from Sinopharm Chemical Reagent Co., Ltd. (Shanghai, China). Deionized water was purified by a microporous water purification system.

### 4.2. Synthesis of Amphoteric Monomer 4-VPPS

The 4-Vinylpyridine (0.1 mol, 10.51 g) was added to a 250 mL round-bottom flask containing 150 mL benzene as the solvent, followed by the addition of 1,3-propanesultone (0.11 mol, 13.43 g). The reaction was carried out at 80 °C for 20 h. After completion, the mixture was filtered to obtain an off-white solid. The product was washed repeatedly with acetone to remove impurities. Finally, the off-white powdered product (4-VPPS) was obtained by vacuum-drying at 50 °C for 24 h and stored sealed under refrigeration for future use, with a yield of approximately 89%.

### 4.3. Preparation of Polyacrylamide Emulsions (PolyAMASV)

We dissolved 10 g of AMPS in 40 g of deionized water, then added 0.1 g of V-50 initiator, and reacted at 50 °C under a nitrogen atmosphere for 6 h to obtain a 20 wt% dispersion stabilizer pAMPS for later use. Then, we dissolved 8 g of AM, 2 g of AMPS, 0.5 g of AA, 0.085 g of SMA, and 10 g of the dispersion stabilizer pAMPS solution in 50 mL of a 25 wt% ammonium sulfate solution. Subsequently, we controlled the amount of VPPS, based on the total mass of the polymerization monomers (1.5%, 1.8%, 2.3%, 2.7%). After thorough stirring, we added 1 g of OP-10 and 0.5 g of dodecyl mercaptan, respectively. We adjusted the pH to 5 using NaOH. We purged it with nitrogen for 30 min to remove oxygen, then heated to 50 °C and added the V-50 initiator. After reacting for 6 h, polymer emulsions were obtained (named polyAMASV-1 to polyAMASV-4, respectively).

### 4.4. Preparation of Copolymers Containing SBMA, DVBAPS, and VBIPS

We dissolved 10 g of AMPS in 40 g of deionized water, then added 0.1 g of V-50 initiator, and reacted at 50 °C under a nitrogen atmosphere for 6 h to obtain a 20 wt% dispersion stabilizer pAMPS for later use. Then, we dissolved 8 g of AM, 2 g of AMPS, 0.5 g of AA, 0.085 g of SMA, and 10 g of the dispersion stabilizer pAMPS solution in 50 mL of a 25 wt% ammonium sulfate solution. Subsequently, SBMA/DVBAPS/VBIPS with a total monomer mass of 1.8 wt% was added. After stirring evenly, 0.02 wt% OP-10 and 0.01 wt% dodecyl mercaptan were added, respectively. We adjusted the pH to 5 with NaOH. After purging with nitrogen for 30 min to de-aerate, we raised the temperature to 50 °C and added V50 initiator. The reaction lasted for 6 h, and finally the polymer emulsions polyAMAS-SBMA/polyAMAS-DVBAPS/polyAMAS-VBIPS were obtained.

### 4.5. Characterization

The chemical structure of the polymer was characterized by Fourier-transform infrared spectroscopy (FT-IR, Bruker Vector 22 from Bremen, Germany), with a scanning range of 4000–400 cm^−1^, a resolution of 4 cm^−1^, and 32 cumulative scans. ^1^H nuclear magnetic resonance (NMR) spectra were recorded on a Bruker Avance III (from Germany) 500 MHz spectrometer using D_2_O as the solvent, with 32 scans and a relaxation delay of 1 s. The sample preparation was as follows: We used 10 mg of the polymer sample and dissolved it in 0.6 mL of deuterated reagent for testing. X-ray diffraction (XRD) analysis was performed on an Ultima IV (from Rigaku Corporation, Tokyo, Japan) diffractometer (Cu-Kα radiation, λ = 0.1545 nm, 40 kV/40 mA) at a scanning rate of 20°/min (2θ range: 10–80°). The sample preparation was as follows: The polymer was freeze-dried and then ready for testing. Thermal stability was evaluated by thermogravimetric analysis (TGA, DMA Q800) under a nitrogen atmosphere (flow rate: 60 mL/min) with a heating rate of 10 °C/min (40–600 °C) The sample preparation was as follows: We weighed 8 mg of polymer powder for testing. The particle size distribution of the emulsion was analyzed using a Zetasizer Ultra (from Malvern Panalytical, Shanghai, China), and the microscopic morphology was observed by scanning electron microscopy (SEM, FEI Nova Nano 450 from FEI Company, Hillsboro, OR, USA).

### 4.6. Rheological Performance Test

The rheological properties of the polymer were investigated using a torque rheometer (HR20 from TA, Santa Fe Springs, CA, America). A shear rate sweep test (0.1–1000 s^−1^) was conducted to evaluate viscosity variations. Shear recovery capability was examined through a three-stage test: initial shearing at 170 s^−1^ for 200 s, followed by high-rate shearing at 1022 s^−1^ for 200 s, and finally returning to 170 s^−1^. Temperature resistance was assessed by monitoring viscosity changes during heating (4 °C/min to 80 °C) and isothermal holding (200 s at 170 s^−1^). Viscoelastic characteristics were determined via frequency sweep (0.1–100 Hz) at a constant strain of 0.1%, measuring both storage modulus (G′) and loss modulus (G″).

### 4.7. Sand-Carrying Performance Test

We prepared the polymer solutions with different salt types, mixed thoroughly with 70/140 mesh quartz sand, and quickly transferred to a settling cylinder. We allowed them to stand at a constant temperature and recorded the time required for the quartz sand to settle completely.

### 4.8. Fracturing Fluid Break Test

The polymer aqueous solution was mixed with gel breaker and maintained in an 80 °C water bath for 4 h, after which its viscosity and surface tension were measured. The broken solution was then transferred to a centrifuge tube and its initial mass recorded, with an equal mass of deionized water set as the control. Following centrifugation at 8000 r/min for 5 min, the sample was dried to a constant weight; the residue content was calculated based on the mass difference.

## Data Availability

The original contributions presented in the study are included in the article, further inquiries can be directed to the corresponding authors.
